# Incretins and SGLT-2 inhibitors in diabetic patients with neuroendocrine tumors: current updates and future directions

**DOI:** 10.1007/s11154-025-09958-5

**Published:** 2025-04-02

**Authors:** Rosaria M. Ruggeri, Erika Maria Grossrubatscher, Eleonora Ciocca, Iderina Hasballa, Simona Jaafar, Monica Oldani, Manila Rubino, Flaminia Russo, Andrea M. Isidori, Annamaria Colao, Antongiulio Faggiano, on behalf of NIKE group

**Affiliations:** 1 Endocrinology, Department of Human Pathology of Adulthood and Childhood DETEV, University of Messina, Messina, Italy; 2 Endocrine Unit, ASST Grande Ospedale Metropolitano Niguarda, Milan, Italy; 3 Department of Experimental Medicine, Sapienza University of Rome, Rome, Italy; 4 Endocrinology Unit, Department of Internal Medicine and Medical Specialties (DIMI), University of Genoa, 16132 Genoa, Italy; 5 Department of Biomedical Sciences, Humanitas University, Pieve Emanuele, Milan, Italy; 6 Endocrinology, Diabetology and Andrology Unit, IRCCS Humanitas Research Hospital, Rozzano, Milan, Italy; 7 Laboratory of Geriatric and Oncologic Neuroendocrinology Research, IRCCS, Istituto Auxologico Italiano, Milan, Italy; 8 Onco-Endocrinology Unit, European Institute of Oncology, Milan, Italy; 9 Endocrinology Unit, Department of Clinical and Molecular Medicine, European Neuroendocrine Tumor Society (ENETS) Center of Excellence, Sapienza University of Rome, Sant’Andrea University Hospital, Rome, Italy; 10 Endocrinology, Diabetology and Andrology Unit, Department of Clinical Medicine and Surgery, Federico II University of Naples, Naples, Italy; 11 UNESCO Chair “Education for Health and Sustainable Development”, Federico II University, Naples, Italy

**Keywords:** Neuroendocrine tumors, Diabetes, GLP-1R agonists–DPP-4 inhibitors, SGLT-2 inhibitors, Dual GIP/GLP-1R agonists

## Abstract

Neuroendocrine tumors (NET) are frequently associated with glycemic disorders, such as prediabetes or diabetes, which may result from either surgical or medical treatments or hormonal hypersecretion by the tumor itself. Moreover, pre-existing diabetes is a known risk factor for NET development, with metabolic control and antidiabetic therapies potentially influencing tumor progression. The complex interplay between diabetes and NET, which share several molecular pathways, has spurred interest in the anti-cancer effects of antidiabetic medications. This is particularly relevant as new antidiabetic drugs continue to emerge, including sodium-glucose cotransporter-2 (SGLT2) inhibitors and incretin-based therapies, such as dipeptidyl peptidase-4 (DPP-4) inhibitors, glucagon-like peptide-1 receptor (GLP-1R) agonists and dual GIP/GLP- 1 R agonists. This review explores the impact of these novel pharmacological options on NET development and progression through a comprehensive analysis of pre-clinical and clinical studies, with the purpose to evaluate safety and feasibility of introducing these drugs in the treatment of NETs patients. We conducted a comprehensive search of online databases, including PubMed, ISI Web of Science, and Scopus, for studies assessing the therapeutic effects and potential mechanisms of action of incretins and SGLT2 inhibitors in patients with NET. These novel antidiabetic drugs exhibit promising anticancer properties, potentially inhibiting tumor cell proliferation and inducing apoptosis, though concerns about certain cancer risks remain. Based on current evidence, the benefits of incretin-based therapies outweigh any potential cancer risks, leading to the proposal of tailored management algorithms for diabetes in NET patients, factoring in the diabetes aetiology, comorbidities, and life expectancy.

## Introduction

### Diabetes and neuroendocrine tumors: two diseases closely linked

Neuroendocrine neoplasms (NENs) are a heterogeneous group of tumors originating from cells with both neural and endocrine properties. These tumors primarily arise in the gastro-entero-pancreatic tract (GEP-NENs) but can also develop in the bronchopulmonary system and other body areas, and they frequently produce peptide hormones or biogenic amines leading to functional syndromes [1]. In most cases, these are well-differentiated, low-proliferating neoplasms, called neuroendocrine tumors (NETs), to distinguish them from poorly differentiated, highly proliferating neoplasms, called neuroendocrine carcinomas (NECs) [2]. While NECs are aggressive, fast-growing neoplasms with a reduced life expectancy, NETs often display and indolent and slowly progressive course, and prolonged survival. Given this markedly different biological behavior, the management of the two conditions is generally distinct, with the often-poor prognosis of NECs leaving little to no room for addressing patients' comorbidities, such as diabetes.

The incidence of NETs has been steadily increasing over the last decades [1, 3], in parallel with the increasing prevalence and incidence of diabetes mellitus (DM) [4], and the two conditions strongly associate and influence each other [5–9]. On the one hand, NETs can increase the risk of developing DM, which may result from either surgical or pharmacological treatments for NETs or from hormonal hypersecretion by the tumor itself [5–7, 10]. On the other hand, several studies have identified DM as an independent risk factor fot the development of GEP-NETs, particulary those of pancreatic origin (panNETs) [11, 12]: two meta-analyses concluded that a history of DM is a significant risk factor for panNETs, and that both body mass index BMI and DM are potentially relevant in the development of gastric, pancreatic, and small intestine tumors [13, 14].

Finally, the impact of DM on cancer outcomes remains a topic of debate. Enhanced glucose uptake and utilization are common metabolic abnormalities in human malignancies, providing fuel for the unrestrained growth of cancer cells [15, 16]. Supporting this, some studies have linked glycemic alterations to worse outcomes, including lower progression-free survival (PFS), a higher number of relapses, an increase in distant metastases, lymph node involvement, and reduced overall survival (OS) [6, 7, 17]. Moreover, some evidence on the protective effects of metformin in NET patients has spurred interest in the anti-cancer potential of antidiabetic therapies [12, 18]. This is particularly relevant as new antidiabetic drugs (ADDs) continue to emerge, including dipeptidyl-peptidase 4 inhibitors (DPP-4i), sodium-glucose co-transporter 2 inhibitors (SGLT-2i), glucagon-like peptide-1 receptor agonists (GLP-1RA), and the novel GLP-1R/glucose-dependent insulinotropic polypeptide receptor (GIPR) dual agonist tirzepatide. These newer treatments offer optimal glycemic control with minimal risk of hypoglycemia, promote weight loss, and provide significant benefits for the cardiovascular and renal systems [19–21]. However, their role in an oncological setting has not been fully elucidated. In this review we summarize the state-of-the-art knowledge regarding the potential benefits and harms of these new ADDs in patients with NET, with the intent of providing advice on DM management.

## Search strategy and selection criteria

We searched online databases, including PubMed, ISI Web of Science and Scopus, for publications with the following MeSH terms: “glucagon-like peptide-1 receptor agonists (GLP-1RA)”, “dipeptidyl-peptidase 4 inhibitors (DPP-4i)”, “sodium-glucose co-transporter 2 inhibitors (SGLT-2i)”,“GLP-1R and glucose-dependent insulinotropic polypeptide (GIP) receptor dual agonists “, “semagutide”, “Tirzepatide “, “Diabetes Mellitus”, “anti-diabetic drugs”, each of these terms in combination with “neuroendocrine tumors (NET)”, “pancreatic neuroendocrine tumors”, “gastro-entero pancreatic neuroendocrine tumors”, “lung neuroendocrine tumors”, “bronchial neuroendocrine tumors”, “pheocromocitoma/paraganglioma”, and “cancer”. The retrieved manuscripts were searched for additional relevant references. NECs and their therapies were excluded from our research due to their different biological behavior, the poor life expectancy of these patients, which significantly limits the ability to address their comorbidities, and the numerous complications that may restrict access to treatments other than insulin. Only articles published in English in peer-reviewed journals were included.

## GLP-1RA, GIP/GLP-1R agonists, DPP-4i, and SGLT-2i: friends or foes in nets?

### Data from preclinical studies

**Fig. 1:**
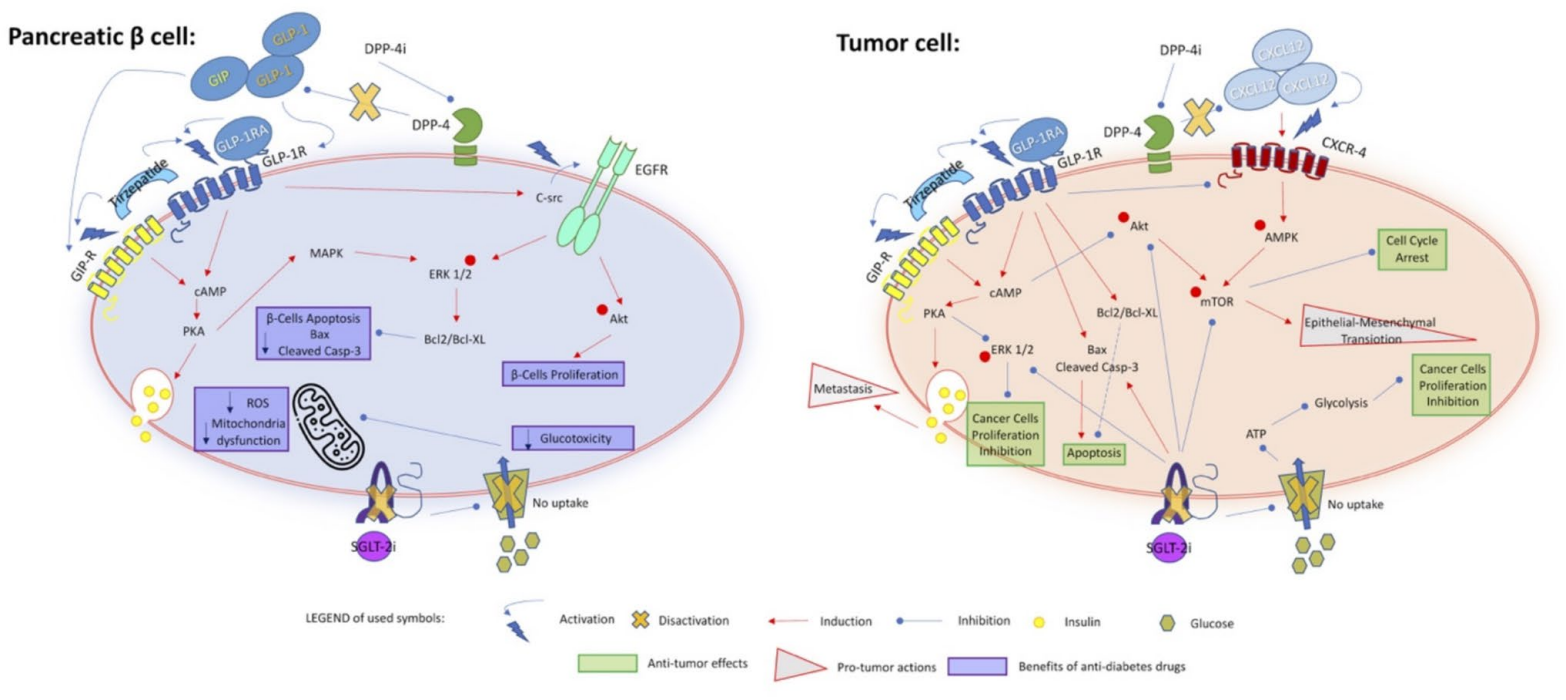
Anti- and Pro-Tumor Mechanisms of GLP-1R Agonists, DPP-4 Inhibitors, and SGLT-2 Inhibitors. The diagram illustrates the main pathways activated or inhibited in pancreatic β cells or tumor cells following treatment with GLP-1/GIP receptor agonists (GLP-1/GIPRAs), DPP-4 inhibitors (DPP-4is), and SGLT-2 inhibitors (SGLT-2is). In the bottom left section, under 'Legend,' the symbols used in the diagram are listed. In summary, in healthy pancreatic β cells, all incretin-mimetic drugs enhance insulin secretion, mitigate glucose-induced cellular damage, inhibit apoptosis, and/or stimulate cell proliferation. GLP-1RAs deactivate GLP-1Rs, leading to the inhibition of the cAMP/PKA/ERK1/2 signaling cascade and tumor growth while promoting apoptosis by increasing levels of pro-apoptotic proteins. DPP-4is activate the CXCL12/CXCR4 pathway, which influences AMPK and mTORsignaling, potentially resulting in an increase in epithelial-mesenchymal transition (EMT). SGLT-2is reduce glucose uptake, inhibit glycolysis, and limit ATP production, thereby contributing to decreased cancer cell proliferation

The activation of GLP-1 receptors, directly by GLP-1RAs or indirectly through reduced degradation of GLP-1 via DPP-4i, has an important role in the functional activity of islet-cells, controlling the secretion of insulin, glucagon, and somatostatin, and thus facilitating glucose disposal. Besides, incretins provide significant benefits to pancreatic β cells by activating specific intracellular pathways that enhance their function, promote survival preserving the β cell mass, and mitigate stress [22, 23] (Fig. 1). DPP-4i, by prolonging the half-life of endogenous incretins such as GLP-1 and GIP, reinforce these pathways, further supporting the preservation and regeneration of the β-cell mass [24](Fig. 1). Similarly, SGLT2i, by reducing glucose reabsorption in the kidneys and mitigating hyperglycaemia, alleviate glucotoxicity and oxidative stress in β cells (Fig. 1).

The pro-proliferative and anti-apoptotic effects of incretins on pancreatic islets have raised concerns about potential risks of inappropriate cell proliferation and neoplastic transformation [25]. This concern for cancer risk stems from *in vitro* and animal studies [26–40]. As summarized in Table 1, these studies were conducted on different cell lines and in different animal models, using both GLP-1 or its analogues or DPP-4i. GLP-1 and its analogue exendin-4 were able to promote β-cell proliferation and survival by activating cAMP and PI3K pathways, involving also upregulation of anti-apoptotic proteins like Bcl-2 and Bcl-xL, [25, 41–43] and regulate key molecular pathways involved in inflammation, tumor cell growth, and oxidative stress [44] (Fig.1). Moreover, the inhibition of glucagon signalling may lead to α cell hyperplasia, that represents a possible precursor lesion of panNET in animal models of glucagon receptor-deficient mice [45]. However, a 2-year toxicity study showed that the GLP-1RA liraglutide did not increase pancreatic adverse events nor induce structural pancreatic changes in mice, rats, or monkeys [46]. Subsequent studies demonstrated that pancreatic histology was not altered by 18 weeks of GLP-1RA treatment in young mice [47].

**Table 1:** Proliferative and antiapoptotic actions of incretin-based therapies on pancreatic islets in animal models and in *vitro* studies

Author (Year)[REF]	Experimental model	Drug used	Main findings
Buteau et al. (1999) [26]	INS-1 cells	GLP-1	GLP-1 acts as a growth factor for β cells by a PI3K mediated event
Xu et al. (1999) [27]	Adult rats previously subjected to subtotal pancreatectomy	Exendin-4	Exendin-4 stimulates differentiation of β-cells from ductal progenitor cells (neogenesis) and proliferation of β-cells
Perfetti et al. (2000) [28]	Young (6 months) and old (22 months) Wistar rats	GLP-1	GLP-1 induces pancreatic cell proliferation and β-cell differentiation through up-regulation of Pdx1 expression
Farilla et al. (2002) [29]	Zucker diabetic rats	GLP-1	GLP-1 provides an increase in islet cell proliferation and a decrease of cellular apoptosis
Wang and Brubaker (2002) [30]	db/db mice	Exendin-4	Exendin-4 increases β -cell mass, proliferation and decreases apoptosis
Li et al. (2003) [31]	Wild-type C57BL/6 mice	Exendin-4	Exendin-4 inhibits streptozotocin induced apoptosis
Hui et al. (2003) [32]	MIN6 cells	GLP-1	GLP-1 inhibits apoptosis of cells exposed to hydrogenperoxide, a proapototic stimulus, in a cAMP and PI3K dependent manner, in association with up-regulation of Bcl-2 and Bcl-xl and reduced poly-(ADP-ribose) Polymerase
Farilla et al. (2003) [33]	Freshly isolated human islets	GLP-1	GLP-1 preserves morphology and function and inhibits cell apoptosis by up-regulation of bcl-2 and down-regulation of the intracellular levels of the active form of caspase-3
Buteau et al. (2004) [34]	INS-1 cells	GLP-1	GLP-1 has a protective effect on β cell gluco-, lipo- and glucolipotoxicity by via protein kinase B activation and possibly its downstream target nuclear factor-κB
Wang et al.(2004) [35]	INS-1 cells	GLP-1	GLP-1 stimulates cell proliferation in a dose-dependent manner and inhibits staurosporine-induced apoptosis by activation of protein kinase B
Kang et al. (2006) [36]	INS-1 cells isolated from pancreatic islets of Sprague–Dawley rats	Exedin-4	Exendin-4 regulates β-cell proliferation by an increased expression of cyclin D1 gene
Cornu et al. (2009) [37]	MIN6 cells and primary islets	GLP-1	GLP-1 protects β –cells against apoptosis by enhancing the activity of an IGF-2/IGF1-receptor autocrine loop
Inaba et al. (2012) [38]	Non-obese type 2 diabetic Goto-Kakizaki rats	Vildagliptin	Vildagliptin enhances β and αcell proliferation, and increases the number of small neogenetic islets
Vrang et al. (2012) [39]	Zucker diabetic fatty rats	Exenatide and liraglutide	Exenatide and liraglutide increase β-cell mass and proliferation
Chen et al. (2013) [40]	INS-1 cells in high glucose conditions	Liraglutide	Liraglutide increases β-cell viability in high glucose conditions by reducing β-cell apoptosis and increasing autophagy by cAMP

*GLP-1R* Glucagon-like peptide-1 receptor; *INS-1 cells* rat pancreatic β cell line; *MIN6 cells* mouse insulinoma cell line; *PI3K* phosphatidylinositol 3-kinase; *Pdx1* pancreatic duodenum homeobox-1; *cAMP* cyclic adenosine monophosphate

On the other hand, incretins displayed anti-cancer potential in different settings, including hepatocellular carcinoma, breast, prostate and pancreatic cancers [44, 48–50]. In a study on pancreatic cancer models, liraglutide exhibited anti-tumor effects on gemcitabine-resistant human pancreatic cancer cells. Moreover, it enhanced the therapeutic efficacy of gemcitabine by modulating the NF-κB signalling pathway [51]. These findings suggest that GLP-1RA may be safe treatments for patients with pancreatic cancer and DM, particularly for those with gemcitabine resistance [52]. In the setting of NET, liraglutide seems to be beneficial in treating panNETs by inhibiting *in vivo* and *in vitro* the PI3K/AKT pathway with a reduction of metastasis, and cancer growth [52, 53]. Specifically, these studies, using a model pancreatic/neuroendocrine cell line PANC-1, have suggested that the GLP-1R activation lead to an increase in cAMP production that is able to inhibit the AKT phosphorylation. At the same time, liraglutide increased the pro-apoptotic proteins Bax and cleaved Caspase-3 levels [53]. Zhao et al. reported that liraglutide inhibited cancer growth and metastasis and increased apoptosis in human pancreatic cancer cell lines and a mouse xenograft model via the inhibition of PI3K/AKT and ERK1/2 signaling pathways [54]. In prostate/neuroendocrine cell lines, exendin-4 has demonstrated to modulate ERK/MAPK pathway attenuating prostate cancer growth, but it was not able to influence apoptosis [49]. In conclusion, GLP-1RAs demonstrated the potential to influence tumour cell proliferation, apoptosis, and angiogenesis, and the overall impact of their effects appears to be highly dependent on the specific type of tumour, its mutational profile and its microenvironment [49, 55–60]. Whether tirzepatide could have similar properties through the activation of the GLP-1R/ GIPR is yet to be determined. On the one hand, it is conceivable that tirzepatide may exert ant-cancer effects, as previously demonstrated for other incretins. On the other hand, in the pancreatic β-cell tirzepatide is able to promote the synthesis of insulin, insulin-like growth factors (IGF) 1 and 2, and IGF-binding protein 3, that in turn activates key pathways, including PI3K/AKT/mTOR, Ras-Raf-ERK, MAPK and JAK-STAT, in target cells [61, 62]. The aberrant activation of these signalling pathways in an individual with a prior history of pancreatitis could increase the risk of developing pancreatic cancer [63–66].

Similarly, DPP-4i has been shown to act either as a promoter or as a suppressor for cancer [67, 68] (Fig. 1). The DPP-4 enzyme, called also CD26, is involved in T cells activation, and has a role in regulating inflammation and concentrations of various chemokines, including SDF-1. Preclinical data suggested that DPP-4i may promote the progression of pre-existing cancer, favouring metastatic spread, epithelial-mesenchymal transition (EMT) and activation of nuclear factor erythroid 2-related factor 2 (NRF2) [69]. Nevertheless, inhibition of CD26/DPP4 suppressed growth in human lung adenocarcinoma cell line by enhancing pro-inflammatory activity of macrophages and NK cell cytotoxicity [70]. Moreover, DPP-4i could have anti-cancer effects by modulating the DPP-4/SDF-1/CXCR4 axis and the immune balance, enhancing tumor rejection by preserving biologically active CXCL10 and increasing trafficking into the tumor by lymphocytes [71]*.* An in vitro study demonstrated that CD26 is expressed on the surface of rat insulinoma cells. The DPP-4i sitagliptin suppressed NF-κB activation and expression of inflammatory cytokines, and reduced cell apoptosis in rat insulinoma cells, suggesting that DPP-4i may exert direct anti-inflammatory effects in islet β cells [72]. Based on this data, these drugs could have a beneficial effect for patients suffering from NETs overexpressing DDP-4. Unfortunately, studies on pancreatic cancer have not shown any real benefit from the use of these drugs; the conclusions are rather neutral [73–77], and no data are available in NET.

Also SGLT-2i, that promote urinary excretion of glucose blocking the SGLT-2 protein, have been demonstrated to stimulate cell proliferation and inhibit apoptosis in pancreatic islets, leading to a preservation of β -cell mass and function in preclinical studies (Table 2) [78–83]. As concerning cancer risk, a possible association between SGLT-2i and cancer has been reported in rats and mice. In particular, a two-year rat study revealed that high dose of canagliflozin could be responsible for renal tubular tumors and pheochromocytomas, due to its effect on carbohydrate malabsorption evidenced by the inhibition of intestinal glucose uptake, decreased intestinal pH and increased urinary calcium excretion. However, these suggested mechanisms are not considered relevant to humans [84, 85] On the other hand, several studies have suggested potential indirect benefits of SGLT-2i in reducing tumor growth by altering its metabolic environment [86]. Indeed, considering that many tumors exploit glucose and glycolysis as an energy source, as it happens in various NETs that overexpressed GLUT or SGLT proteins, the inhibition of glucose uptake can lead to a decrease in ATP production and cell growth [87]. The direct anti-cancer effects of these drugs is based on various mechanisms (Fig. 1). For instance, in hepatocellular carcinoma, these drugs induce in vitro cell cycle arrest and promote apoptosis increasing the levels of cleaved Caspase -3 [86]. Another critical mechanism is the inhibition of the PI3K/AKT/mTOR signaling pathway, that is well known to be crucial also in NET tumorigenesis (Fig. 1). By inhibiting this pathway, SGLT-2i reduce cell growth and can potentially enhance the efficacy of existing cancer therapies, also in NETs. As seen in the PC-3 cells model, SGLT-2i can also activate AMP-activated protein kinase (AMPK), which plays a central role in the suppression of fatty acid synthesis and the downregulation of proteins involved in cell proliferation, such as SREBP1 and SCD1 [88].

**Table 2:** Proliferative and antiapoptotic actions of SGLT2 inhibitors on pancreatic islets in animal models and in *vitro* studies

Author (Year)[REF]	Experimental model	Drug used	Main findings
Nagata et al (2013) [78]	Female db/db mice	Tofogliflozin	Tofogliflozin preserves pancreatic β-cell function, prevents the decrease of plasma IRI levels and results in a significant increase in IRI levels, compared with the untreated group at 8 weeks of treatment
Terami et al (2014) [79]	Male db/db mice	Dapagliflozin	Dapagliflozin significantly prevents the decrease in β-cell mass in a dose-dependent manner
Okauchi et al (2015) [80]	Obese T2DM db/db mice	Luseogliflozin	Luseogliflozin increases β-cell mass by both increasing beta-cell proliferation and reducing beta-cell apoptosis
Takahashi et al. (2018) [81]	db/db mice of different ages (aged 6, 10, 14 and 24 weeks old)	Luseogliflozin	Luseoglifozin significantly increases β-cell mass compared with the control group regardless of age
Kanno et al (2018) [82]	T2DM db/db mice	Dapagliflozin	Dapagliflozin increases islet and β cell numbers up-regulating expression of Agr2, Tff2 and Gkn3
Wei et al (2020) [83]	Cultured primary rodent islets and αTC1 clone 9 cells in high glucose conditions	Dapagliflozin	Dapagliflozin enhances β cell self-replication, induces α to β cell conversion, and promotes duct-derived β cell neogenesis, up-regulating the expression of pancreatic endocrine progenitor and β cell specific markers (including Pdx1). The promoting effects of dapagliflozin on β cell regeneration may be partially mediated via GLP-1 secreted from α cells

*SGLT2* Sodium-glucose cotransporter-2, *T2DM* Type 2 diabetes mellitus, *IRI* immunoreactive insulin, *Agr2* Anterior gradient protein 2 homolog, *Tff2* Trefoil factor 2, *Gkn3* Gastrokin 3, *Pdx1* pancreatic duodenum homeobox-1, *GLP-1* glucagon-like peptide-1

### Data from human pathology and clinical studies

Data regarding a theoretical risk of NETs are scanty and limited to panNETs. This is not surprising considering the fact that normal pancreatic islets express the GLP-1R [89], and the GLP-1R has been demonstrated to be overexpressed in some panNETs, mainly insulinomas, with contrasting evidence in metastatic lesions [90–94]. GLP-1 receptors are also overexpressed in NETs of gut and lung origin, but no evidence of any causal correlation has been documented between GPL-1RA therapies and these NETs [91]. Also the GIPR is expressed in several normal tissues, including pancreas, bones, brain, and adipose tissue [95], and its expression was found significantly higher in functional NETs (including insulinomas and gastrinomas), and non-functional panNETs, compared to non-neoplastic tissue [61].

Only one study evaluated pancreatic pathology with regards to incretin therapies in humans [96]. Butler and colleagues evaluated the pancreatic pathological changes in three groups of brain-dead organ donors: non-diabetic (n = 14), DM without incretin therapies (n = 12) and DM with incretin therapies for 1 year or more [96]. The authors observed that incretin-treated patients showed marked expansion of both the exocrine and endocrine pancreas, α-cell hyperplasia, glucagon-expressing microadenomas in 3 cases and a NET in one case, suggesting a correlation between incretin treatment and panNETs in humans [96]. However, the study has several limitations including the small samples size, the not matched baseline characteristics of incretin users and non-users, and the lack of evidence of previously tumor-free pancreata [97–100]. In fact, postmortem studies suggest that silent panNETs are quite common (up to 10% of older people) [101]. Moreover, DM is more common in patients with panNETs than in controls [102], so it is conceivable to expect that some DM patients taking incretins will be diagnosed with a panNET.

Overall, findings in clinical human studies regarding the possible association between incretin-based therapies and panNETs are controversial and largely inconclusive. A higher risk of pancreatic cancer, also of neuroendocrine origin, was reported for GLP-1RA in several analyses of the Food and Drug Administration adverse events reporting system (FAERS) database. In the study by Yang et al. disproportionally analysis revealed that GLP-1RA showed an increased risk of thyroid, pancreatic and islet cell neoplasms and APUDoma NEC [103]. Similarly, an increased risk of the class “pancreatic cancer”, including also panNETs, was reported by Wu et al. and Cao et al. [104, 105]. However, although based on data from a large number of cases, these observational studies have the limitation that extract data from the FAERS database do not prove causality. In addition, these databases rely on voluntarily reported information suffering from reporting bias, and analysed data also do not consider established risk factors such as smoking, family history, obesity, alcohol consumption and other concomitant medications.

Similarly, a higher risk of pancreatic cancer could not be demonstrated in the numerous observational studies in humans and analyses of data from clinical trials. Although this potential adverse event needs to be monitored long-term due to the latency of cancers, the overall findings provide some reassurance about the safety of incretins. Several meta-analyses focused on the evaluation of neoplastic risk in patients treated with incretins failed to report an increased risk of pancreatic cancer [106, 107]. In the most important prospective randomized controlled trials primarily evaluating the cardiovascular safety of incretin-based therapy, an increased risk of pancreatic cancer was not observed in patients treated with incretins compared to controls [108, 109]. Unfortunately, in most studies included in the meta-analysis, it is generally not known whether panNETs are included among pancreatic cancers. Noteworthy, the only neuroendocrine pancreatic tumour reported in the SAVOR-TIMI study was observed in the placebo arm [110]. With regards to cancers other than pancreatic and thyroid cancer, available studies supported a neutral association with incretin-based therapies in humans [111].

Finally, the safety profile of tirzepatide has been evaluated in several trials in people with obesity (the SURMOUNT-1 trial), with DM (SURPASS clinical trials), and with both obesity and DM (SURMOUNT-2 trial). These studies did not record any cases of medullary thyroid or pancreatic cancer [112–114]. Moreover, a recent meta-analysis of available published randomized trials suggests that tirzepatide may not affect the risk of malignancy [115]. Substantial body weight reduction seen with tirzepatide use in individuals with DM and obesity could be of paramount importance in the long-run in terms of reduction in the risk of cancer, based on previous findings from the Look AHEAD trial, which demonstrated a 16% reduction the incidence rates of obesity-related cancers in individuals with concomitant DM after intensive body weight loss, over a median follow-up of 11 years in total [115]. Nevertheless, tirzepatide is not recommended in patients who have had pancreatitis and with medullary thyroid carcinoma or if the patient has multiple MEN2 [116].

Regarding DPP-4i, a single case report from Pech et al. described an increase in serum serotonin levels in a patient with metastatic carcinoid, after the initiation of saxagliptin, with subsequent normalization after its suspension. However, no data in the literature has demonstrated so far any relationship between DPP-4i and NET development or progression in humans [117].As concerning SGLT-2i, the correlation between their use of and the possible occurrence of tumors in patients, especially on short-term monitoring, seem to be largely inconclusive [118, 119].Up to date, available data from randomized trials do not suggest a detrimental effect of SGLT-2i on the incidence of malignancies in general, or in NETs in particular [120].Despite the promising preclinical data on these drugs in different oncological settings, the low number of studies on their mechanism of action in NET represents a significant opportunity to explore their use as a new adjuvant therapy, especially given their minimal toxicity [121].

## Role of metabolic control in reducing aggressiveness of net and improving overall prognosis: clinical studies

Few clinical studies are available about the effect of metabolic control on the outcomes of NETs patients, and available data are conflicting (Table 3).

**Table 3:** Clinical studies investigating association between metabolic control and prognostic variables in NETs

Author	Study population	Main findings
Sandini 2020 et al. (2020) [122]	Prospective study of 417 patients underwent to pancreatic resection for panNETs	Patients with dysglycemia had greater rates of metastasis, vascular, perineural, and lympho-vascular involvement Preoperative dysglycemia was associated with impaired OS (HR 1.57, 95% CI 1.01–2.46) and RFS (HR 1.78, 95%CI 1.01–3.12)
Gong et al (2021) [123]	Retrospective study of 201 patients with not pre-existing DM with radically resected panNETs	High FBG levels were significantly associated with poor OS (OS; p = 0.019) and RFS (RFS; p = 0.011)
De Mestier et al (2020) [124]	Retrospective study of 268 patients with or without DM underwent surgical resection of non-metastatic PanNETs	DM exacerbation was independently associated with an increased risk of PanNETs recurrence (HR 2.35, 95% CI [1.24–4.47], p = 0.009)
Capurso et al (2009) [17]	Case–control study of 162 PanNETs and 648 controls	A recent diagnosi of DM (< 12 months) is an independent risk factor for panNETs (OR 40.1, 95% CI 4.8–328.9)
Pusceddu et al (2018) [18]	Retrospective study of 445 patients with advanced PanNETs treated at 24 medical centers in Italy	PFS was significantly longer in patients with DM than without DM (median 32 vs 15 months, p = 0002; HR for patients with vs. without diabetes, 0.63) PFS of patients treated with metformin was significantly longer than for patients without DM and patients with DM receiving other treatments
Pusceddu et al (2021) [126]	Post-hoc analyses of phase III CLARINET study. 204 patients with advanced GEP NETs treated with lanreotide 120 mg. Seventy-nine patients with DM and 125 without DM	No differences in PFS between patients with and without DM (hazard ratio 1.20 {95% confidence interval 0.79 to 1.82}; *p* = 0.380). No difference in PFS was observed in lanreotide-treated patients with and without DM (*p* = 0.8476)
Pusceddu et al (2023) [133]	Phase Ib trial in 20 patients with and without DM, with advanced GEP NETs (18 patients) and thoracic NETs (2 patients) treated by metformin and lanreotide autogel	Therapy with metformin and lanreotide is safe and well tolerated (5% incidence of SAEs) No statistically significant PFS differences between DM and non DM patients
Glasberg et al (2022) [127]	Single-arm phase II trial in 28 patients with progressive metastatic GEP (24 patients) or pulmonary NETs (4 patients) treated with metformin	Twenty-six patients had progression, with 13 (46%) presenting DCR at 6 months and a median PFS of 6.3 months (95% confidence interval: 3.2–9.3) There was no objective response
Hue-Fontaine et al (2020) [128]	Retrospective study in 213 patients (197 patients with GEP NETs and 35 patients with lung NETs), 165 without DM; 19 with DM treated with metformin and 29 with DM treated with others anti-diabetic drugs. 197 patients	No significant difference in median PFS was found between the three groups
Kusne et al (2021) [9]	Retrospective study in 118 patients with newly diagnosed NETs (87 patients with GEP NETs, 20 patients with lung thymus NETs 8 patients unknow primary) with and without DM and 59 matched pairs	Three-year overall survival was 72% (95% CI: 60–86%) for DM patients versus 80% (95% CI: 70–92%) for non-DM patients (p = 0.82) DM did not adversely affect survival of patients with NET
Umlauft et al (2017) [129]	Cross sectional post hoc analyses on diabetes related outcome in 1535 NETs patients enrolled in a prospective trial with RLT	After treatment, 72 patients developed DM OS was similar in patients with and without DM (hazard ratio, 1.13; 95% confidence interval, 0.91–1.39; n = 1,535; P = 0.27)

Dysglycemia: blood glucose ≥ 140 mg/dl and/or HbA1 ≥ 6.5%. *FBG* fasting blood glucose. *DCR* disease control rate

Some studies addressed the association between overall prognosis and glycemic alterations in patients undergone surgery for panNETs. Sandini et al. found that patients with preoperative impaired glucose metabolism (blood glucose ≥ 140 mg/dl and/or HbA1 ≥ 6.5%) had reduced overall survival (OS) and recurrence free survival (RFS), with greater rate of metastasis and vascular, perineural and lymph-node invasion, compared to patients with normal blood glucose. Surprisingly, no differences were found between diabetic and non-diabetic patients before surgery [122]. Similar results were showed by Gong et al. in a retrospective study on 164 patients without pre-existing DM underwent to resection of non-functional panNETs. Fasting blood glucose > 101 mg/dl was associated with poor OS and RFS [123].

Also post-operative hyperglycemia could impact panNET recurrence in patients undergoing to curative surgery. De Mestier et al. found that DM exacerbation was associated with an increased risk of recurrence regardless age, multifocal disease, grade and TNM stage. Interestingly, metformin use tended to decrease the risk of recurrence [124]. Some evidence is also available in advanced and metastatic GEP-NET and lung/thymus NETs patients. In the study by Capurso et al., recent DM was a risk factor for tumor progression and correlated with higher incidence of metastatic disease at diagnosis [17].

In a retrospective study, Pusceddu et al. analyzed data from over 400 patients with panNETs treated with SSA and or everolimus and investigated the association between glycemia and PFS, and metformin use and PFS. In this study diabetic patients had longer PFS than those without DM (median 32 vs 15 months, p = 0002; hazard ratio for patients with vs. without diabetes, 0.63). Moreover, metformin use was associated with longer PFS compared to both patients without DM and patients with DM treated with other ADDs (not including incretins). However, glucose levels were not associated with PFS in patients with and without DM [125]. Regardless of glycemic control, several other studies have reported a potential anti-tumor role of metformin with prolongation of PFS in GEP-NET and thoracic NETs [126, 127] (Table 3).

In contrast, Hue Fontaine et al. evaluated a series of patients with advanced GEP and lung NETs and failed to find any differences in terms of mPFS between non-diabetic, diabetic patients treated by metformin and diabetic patients treated by other ADDs [128]. Similarly, Kusne et al. found that DM did not affect the survival of patients with GEP and lung/thymus NETs [9]. Moreover, DM did not appear to increase the mortality of NET patients undergoing PRRT [129].

In conclusion, data from the literature are largely inconclusive, and limited to metformin as it concerns the potential antitumor effects of ADDs. Further studies are needed to clarify the prognostic value of metabolic control in diabetic NETs patients and the effect of DPP-4i, SGLT2i, GLP-1RAs and the novel dual agonist tirzepatide on tumor outcomes.

## Net patients with diabetes: how to treat them

Interdisciplinary and patient-centered care is crucial for the management of DM in patients affected by NETs since the interplay between DM, cancer, and antineoplastic therapies is challenging. Considering the detrimental impact of poor glycemic control on the patient’s outcomes, structured clinical guidance is warranted to achieve the anticancer and antidiabetic treatment goals, while minimizing the risk of complications that negatively impact patients’ quality of life (QoL). Due to the lack of specific guidelines, DM management in NET patients should generally conform to the recommendations of the American Diabetes Association (ADA) and the European Association for the Study of Diabetes (EASD) [130]. However, a personalized and careful evaluation is required based on the patient’s overall performance status, life expectancy, comorbidities, NET stage, antineoplastic treatment, and caregivers’ collaboration.

### DM treatment goals, glycemic targets and glucose monitoring

In cancer patients, the primary goal of DM treatment relies on attaining acceptable glycemic levels and optimizing the patient’s QoL. The prioritization of preventing and managing DM acute complications is essential, with a particular emphasis on minimizing the frequency and severity of hypoglycemic events. Despite the focus on short-term goals, preventing DM-related chronic complications should still be considered when reasonable, particularly in patients with longer life expectancy and discreet performance status [131]. Glycemic targets should be tailored to the patient’s status and NET prognosis. Stricter glycemic levels with hemoglobin glycated (HbA1c) ≤ 7%hould be achieved in younger patients with good performance status, longer life expectancy, and initial stages of NET. Higher targets with HbA1c ≤ 8.5% may be acceptable in frail patients with shorter life expectancy and poor NET prognosis.

Indications for self-monitoring blood glucose (SMBG) should adhere to the general recommendations for DM. More frequent monitoring may be warranted in case of acute illness, psychological-physical stress, or therapeutical changes interfering with glucose metabolism. Conversely, in the palliative context, SMBG should be minimized as needed to prevent symptomatic hypo- or hyperglycemia and guarantee major comfort to the patient. In special settings, i.e. high glycemic variability, frequent and/or severe hypoglycemia, or pancreatectomized patients, glucose sensors may be implemented according to the patient’s and caregivers’ preference and local availability.

### Antidiabetic strategies in NET patients

**Fig. 2:**
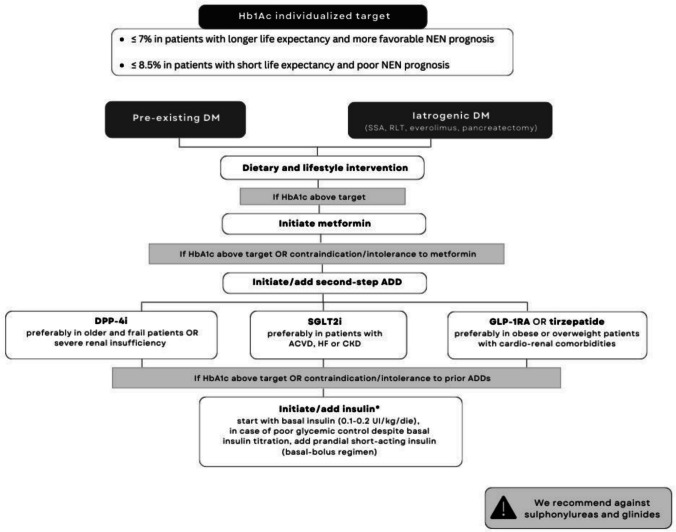
Flow-chart resuming the proposed algorithm for management of pre-existing and/or iatrogenic diabetes in patients affected by neuroendocrine tumors. Abbreviations: DM: diabetes mellitus, NET: neuroendocrine tumours, HbA1c: hemoglobin glycated, SSA: somatostatin analogue, RLT: radioligand therapy, ADD: antidiabetic drug, SGLT-2i: sodium-glucose cotransporter-2 inhibitor, DPP-4i: dipeptidyl peptidase-4 inhibitor

**Fig. 3:**
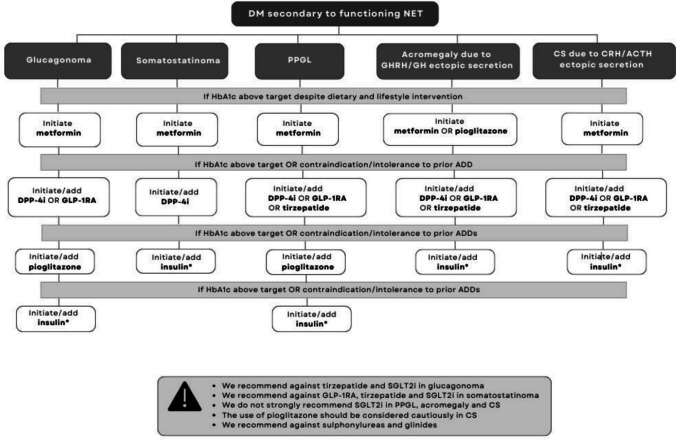
Flow-chart resuming the proposed algorithm for diabetes secondary to functioning NETs. Abbreviations: DM: diabetes mellitus, NET: neuroendocrine tumours, HbA1c: hemoglobin glycated, ADD: antidiabetic drug, SGLT-2i: sodium-glucose cotransporter-2 inhibitor, DPP-4i: dipeptidyl peptidase-4 inhibitor

Antidiabetic strategies should be tailored to each patient according to the causes of hyperglycemia (i.e. pre-existent DM, iatrogenic DM or functioning NETs), the patient’s comorbidities, nutritional status, and prognosis. Pharmacological treatment should be initiated if glycemic values are above the tailored targets despite dietary and lifestyle interventions. Algorithms of DM management in patients affected by NET are proposed (Figs. 2 and 3).

#### Pre-existent and iatrogenic diabetes

In patients with pre-existing DM or iatrogenic DM secondary to anticancer drugs (mTORi, SSAs, radioligand therapy), or sub-pancreatectomy, first-line monotherapy with metformin should be initiated, considering also the potential anti-proliferative effects and synergic interplay with everolimus and SSAs, yet to be further explored and consolidated [18, 126, 132, 133]. If glycemic goals are not achieved with metformin alone, or metformin is contraindicated or not tolerated, a second line ADD should be added or initiated, such as SGLT-2i or DPP-4i. SGLT-2i is a valid alternative for patients developing either impaired insulin secretion or insulin resistance [6]. Given the cardiovascular and nephroprotective benefits, glifozins could be most suitable for NET patients with a history of congestive heart failure or atherosclerotic cardiovascular disease (ASCVD, i.e. myocardial infarction, stroke, peripheral artery disease), as well as in patients with chronic kidney disease (CKD). Such agents reduce the risk of CKD progression; however, their glycemic control decreases with the reduction of the glomerular filtration rate [134, 135].

Regarding DPP-4i, no data in the literature has demonstrated so far any relationship with NET development or progression, so that no specific warning exists. DPP-4i are a viable second step ADD due to their good tolerability and safety profile. Given the lower risk of hypoglycemia, the neutral impact on body weight and the safe use in CKD individuals, these agents are preferable to use especially in older and frail NET patients [136, 137].

As previously reported, the use of incretins is debated in panNETs due to their possible trophic effect on the pancreatic islet [91, 138]. Currently, we lack specific recommendations for or against the routine use of GLP-1RA or tirzepatide in NETs, the only possible contraindication in the neuroendocrine setting being a personal or familial history of MTC or type 2 multiple endocrine neoplasia (MEN2) [139–142]. Considering the favourable cardio-metabolic and nephroprotective benefits, GLP-1RA (liraglutide, subcutaneous semaglutide, and dulaglutide) or tirzepatide could be advantageous second-line options in obese (BMI ≥ 30 kg/m2) or overweight (BMI ≥ 27 kg/m2) patients with additional comorbidities [142, 143]. The prescription of GLP-1RA or tirzepatide, the latter associated with greater weight loss, should be carefully tailored to overweight or obese NET patients with diabetes, characterized by discreet performance status, and no history of weight decrease, cachexia, gastrointestinal disorders or prior pancreatitis.

#### DM secondary to functioning NETs

DM caused by functioning NETs (glucagonoma, somatostatinoma, pheochromocytoma and paraganglioma—PPGL, ectopic GHRH/GH or CRH/ACTH secretion) is generally resolved after cancer surgical removal. However, until surgery or if surgery is not feasible, the first choice ADD is metformin, an insulin sensitizer, aimed at improving the insulin resistance arising from the hypersecretion of counter-regulatory hormones [144]. Pioglitazone could be another first-line insulin-sensitizer in acromegalic patients, considering the correlation between thiazolidinediones and reduced levels of GH and IGF-1 reported in preclinical studies, not confirmed instead in the clinical setting [145, 146]. In case of contraindication, intolerance or failure in achieving the glycemic goals with first-line monotherapy, then second-step ADD should be initiated or added according to the type of functioning NETs (Fig. 3). In glucagonoma the preferable second-line option includes GLP-1RA, alone or combined with metformin, since GLP-1RA inhibits glucagon secretion and enhances insulin production [143]. If GLP-1RA is contraindicated or not well tolerated, DPP-4i could be prescribed. If poor glycemic control persists, pioglitazone may be added as a third-line ADD considering its benefits as an insulin sensitizer [147]. Tirzepatide and SGLT2i should be avoided, due to the secretory effect on glucagon secretion induced by both GIP and glifozins [148, 149].

DM secondary to somatostatinoma is generally mild and controlled with dietary intervention and/or metformin [150]. DPP-4i is a valid second-step alternative to be started or added to metformin. Glifozins should be avoided in patients experiencing diarrhea/steatorrhea, since they could lead to major dehydration. GLP-1RA and tirzepatide are not recommended, considering the clinical spectrum of somatostatinoma (diarrhea/steatorrhea, cholelithiasis, weight loss), as well as the stimulatory effect on pancreatic δ-cells directly induced by GLP1-RA [151].

In PPGL, acromegaly or Cushing syndrome (CS) due to ectopic hormonal secretion, incretin-based therapies should be considered as a second step: either DPP-4i due to their favorable safety profile, or GLP-1RA or tirzepatide due to their cardioprotective effects [147, 152]^.^ In PPGL, GLP-1RA or tirzepatide could be beneficial also considering the reduced GLP-1 production secondary to catecholamine hypersecretion. ^154,160^ If glycemic control is still not optimal or in case of intolerance/contraindication to prior ADDs, a third-line therapy should be started or added. While insulin represents a valid option in acromegalic and CS patients, in PPGL pioglitazone could be evaluated considering its antiproliferative effects reported in pheochromocytoma cells, whereas insulin should represent a last-step therapy in case of poor glycemic control or intolerance/contraindication to pioglitazone [147, 152].

Regarding the use of SGLT2i, their efficacy and safety in PPGL warrants further investigation, since there is evidence that the suppression of SGLT activity may interfere with the functional activity of the pheochromocytoma PC12 cells [153, 154]. In acromegalic patients, glifozins should be evaluated carefully only in suitable cases of appropriate biochemical control of acromegaly, considering the higher risk of diabetic ketoacidosis observed in acromegalic patients [147, 155, 156]. In CS patients the prescription of SGLT2i is not generally encouraged considering the potentially amplified chance of urogenital infections in these subjects [157]. Furthermore, in CS pioglitazone is not strongly recommended due to the major risk of bone loss and fracture, weight gain or edema, which may further compromise the patients’ clinical status [144, 158].

Regardless of DM pathogenesis, the last step ADD includes insulin therapy, which is required in case of total pancreatectomy, severe insulin deficiency, failure to achieve the glycemic goals despite the optimization of previous antidiabetic treatment, intolerance or contraindications to the prior ADDs [159]. According to ADA recommendations, basal insulin should be initiated at 0.1–0.2 units/kg/day, adjusted by 10–15% or 2–4 units once or twice weekly until the target fasting glycemia is achieved. In case of poor glycemic control despite basal insulin titration, basal-bolus regimen should be implemented by adding prandial administration/s of short-acting insulin [160].

Finally, the use of sulphonylureas and glinides is not recommended due to the relevant hypoglycemic risk [6, 144].

### GLP-1RA, DPP-4i, and SGLT-2i safety profile and further considerations

Safety indications on the use of ADDs in patients with NEN are reported in Table 4 [161]. The table also contains specific suggestions on the use of incretins and SGLT-2i in clinical practice.

**Table 4:** Safety of anti-diabetic drugs in patients affected by neuroendocrine tumors

• In case of iodine-based imaging, metformin should be temporarily discontinued at the time of or before the procedure in patients with eGFR 30–60 mL/min/1.73 m^2^, heart failure, hepatic disease, and history of alcoholism
• Metformin, GLP-1RA and tirzepatide should be prescribed cautiously in patients receiving anticancer treatment associated with potential gastrointestinal AEs
• The extended-release metformin formulation should be considered if diarrhea is reported
• Renal function should be monitored periodically, especially in patients receiving potentially nephrotoxic anticancer therapy (i.e. RLT, everolimus, sunitinib, CHT)
• If severe renal insufficiency occurs first choice ADDs include DPP-4i and/or insulin according to the individualized HbA1c target
• ADDs associated with weight loss (i.e. GLP-1RA, tirzepatide) should be avoided in patients with cachexia, inappetence and decreased body weight
• SGLT2i should be avoided in patients prone to diabetic ketoacidosis, recurrent urogenital infections and in elderly patients with poor prognosis
• Tirzepatide and SGLT2i should be avoided in patients with glucagonoma due to the stimulatory effect on glucagon secretion
• GLP-1RA and tirzepatide are not recommended in patients with somatostatinoma due to the stimulatory effect on somatostatin secretion and the potential worsening of gastrointestinal disorders
• SGLT2i should be avoided in patients with somatostatinoma due to the major risk of volume depletion
• SGLT2i is not strongly recommended in patients with ectopic GHRH/GH secretion because of the higher risk of diabetic ketoacidosis
• SGLT2i is not strongly recommended in CS patients due to the higher risk of urogenital infections
• SGLT2i is not strongly recommended in PPGL due the potential interference of the suppressed SGLT activity with the functional activity of PHEO cells
• Pioglitazone should be administered cautiously especially in patients with CS for the higher risk of bone fractures and fluid retention
• The use of sulphonylureas or glinides is discouraged in all patients due to the relevant risk of hypoglycemia

Abbreviations: *eGFR* estimated glomerular filtration rate, *AEs* adverse events, *RLT* radioligand therapy, *CHT* chemotherapy, *HbA1c* hemoglobin glycated, *ADDs* antidiabetic drugs, *GLP-1RA* glucagon-like peptide-1 receptor agonists, *SGLT-2i* sodium-glucose cotransporter-2 inhibitor, *DPP-4i* dipeptidyl peptidase-4 inhibitor, *GHRH* growth hormone-releasing hormone, *GH* growth hormone, *PPGL* pheochromocytoma and paraganglioma, *PHEO* pheochromocytoma, *CS* Cushing’s syndrome

GLP-1RA and tirzepatide should be avoided in patients experiencing inappetence, decreased body weight or cachexia in advanced stages of NETs, and in patients with gastrointestinal adverse events (diarrhea, nausea, vomiting) associated with anticancer therapy (i.e. SSAs, RLT, everolimus, sunitinib, CHT). DPP-4 may be used in patients with renal insufficiency and mild diabetes, in whom metformin is contraindicated as first-line therapy [159]. Regarding SGLT2i, their use is not recommended in patients prone to diabetic ketoacidosis and patients with recurrent urinary and genital infections. Glifozins are generally discouraged in older patients with poor prognosis due to the increased risk of dehydration secondary to osmotic diuresis and urogenital infections, which may further compromise the overall QoL [159].

## Conclusione and perspectives

The therapeutic landscape of DM has dramatically changed in the last decades thanks to the advent of new drugs, including incretin secretory molecules (namely, GLP-1RA, dual GLP-1/GIPR agonist tirzepatide, DPP-4i), and SGLT-2i. These newer anti-diabetic drugs have proven effective in terms of both glycemic control and weight reduction with minimal risk of hypoglycemia, and provide significant benefits in terms of cardiovascular and renal protection. Our understanding of the mechanisms of action and multiple biological effects of these drugs have revealed significant potential in several areas of medical research, ranging from metabolism to neuroprotection. In particular, incretin-based therapies have been studied for their potential in several cancers, since they may regulate key molecular pathways involved in tumorigenesis, such as inflammation, cell growth, and oxidative stress. In this light, the potential for therapeutic intervention in NET patients with DM is particularly intriguing since NET overexpress GLP1-R, and a complex interplay exists between NET, mainly GEP-NET, and diabetic disease. However, safety concerns exist regarding their potential association with pancreatic cancer, mainly based on preclinical studies in animal models. As a consequence, growing attention has been paid to the relationship between these ADDs and cancer development and prognosis, and further research is needed to evaluate the benefits and risks. Based on current evidence, the benefits of incretin-based therapies outweigh any potential cancer risks.

## Data Availability

No datasets were generated or analysed during the current study.
